# How Patients Seek and Value Online Scar-Related Information: A Qualitative Study

**DOI:** 10.3390/ebj7010009

**Published:** 2026-02-06

**Authors:** Koen Maertens, Nancy Van Loey, Peter Moortgat, Jill Meirte

**Affiliations:** 1 Organisation for Burns, Scar Aftercare & Research (OSCARE), 2170 Antwerp, Belgiumpeter.moortgat@oscare.be (P.M.); 2 Clinical and Lifespan Psychology, Faculty of Psychology and Educational Sciences, Vrije Universiteit Brussel, 1050 Brussels, Belgium; 3 Department of Rehabilitation Sciences and Physiotherapy REVAKI-MOVANT, Faculty of Medicine and Health Sciences, University of Antwerp, 2610 Wilrijk, Belgium

**Keywords:** patient-centered care, co-creation, patient and public involvement, patient education, health literacy

## Abstract

Background: Pathological scarring (PS) following surgical procedures, burns, or trauma poses significant clinical, psychological, and socio-economic challenges. Despite the high prevalence of PS, reliable information resources are limited, often leading individuals to depend on unvalidated online sources. To address this gap, we developed MyScarSpecialist.com, an evidence-based website providing comprehensive information on scar types, characteristics, and treatment options. This study aimed to optimize the website through co-creation with patients and clinicians. Methods: Semi-structured focus group meetings were conducted with patients and carers; sessions were recorded, transcribed, and analyzed using thematic analysis. Results: From the 3 focus group meetings with 15 patients with scars and 3 carers, four key themes emerged: (1) Information Sources: The Role of Professionals, Peers, and Digital Media in information sharing; (2) Desired information: From scar typing to treatment outcomes to psychosocial impact; (3) Website design: Audience preferences on content layering, information load, and image positioning; (4) Readability: Optimizing content for comprehension. Participants highlighted the need for enhanced peer support and resources addressing the psychological impact of scarring. Conclusions: These findings provide comprehensive insights for optimizing medical educational websites, ensuring inclusivity, accessibility, and empowerment for patients through co-designed strategies.

## 1. Introduction

Scars may originate from multiple sources and are commonly associated with burns, trauma, surgery, or inflammatory skin diseases [1]. They may significantly impair patients’ quality of life (QOL) [2]. It is well-documented that scars can cause pain, itch, and decreased range of motion [3,4]. In addition, scars can negatively affect appearance resulting in appearance-based stigma, impacting mental well-being and social relationships [5,6,7,8]. Given the potential impact of scars on multiple aspects of health, individuals may have diverse information needs concerning scar-related problems and treatment options.

Each year, about 100 million people seek medical care and information for their scars [1]. Scar management involves both early and late scar interventions. Early treatment, including compression therapy, silicones, mobilizations, taping, laser therapy, and surgical procedures are methods for minimizing scar development [2,4]. In the later stages, scar reconstruction surgery and laser treatments are commonly used interventions [1]. Over the past decade, the amount of online information about scar management has grown rapidly. While this expansion increases access to medical knowledge, the reliability and quality of online content vary widely. A Chinese study examining the quality of scar-related information on TikTok concluded that videos not posted by healthcare professionals were generally of moderate quality [9]. Moreover, patients may find it difficult to recognize credible resources or may lack the skills to evaluate health information critically. Similar challenges have been observed among patients seeking information on gastrointestinal diseases [10]. Furthermore, online information may not be readily understandable to the average patient. Existing guidelines from the American Medical Association [11] recommend that patient educational materials be written at a 6th- to 8th-grade reading level to optimize comprehension. However, this standard is not always met, including in scar information [12]. This indicates that greater efforts are needed to enhance the quality of online scar information sources and for closer alignment of these resources with patients’ needs.

The co-creation of health educational materials with patients has been proposed as an effective way to ensure that health-related information reflects patients’ preferences, needs, and values [13]. Examining patients’ perspectives on online scar-related information can yield valuable insights and uncover blind spots that may inform future research and practice aimed at enhancing website quality and relevance [10]. Including patients’ voices in the development process can ensure that educational materials better address patients’ needs [14]. In Belgium, Oscare, a research and aftercare center specialized in scar treatments, developed the educational website MyScarSpecialist.com. This platform aims to help individuals in accessing reliable information and connect with appropriate healthcare providers. It offers evidence-based resources on scar types and characteristics, presents a range of treatment options, and includes a referral database of healthcare professionals [15]. However, because this website was developed primarily by professionals, it lacks a patient perspective. To better align content with patients’ needs, focus groups involving both patients and experts may offer important insights into how patient preferences can be addressed. Group dynamics within a focus group often help surface the issues that are most salient to the target group and make taboo subjects more accessible. This process can yield valuable insights into participants’ unmet needs [16].

In conclusion, prior studies have documented quality shortcomings in online patients educational materials and stressed the importance of incorporating patients voices into their design [13]. However, within the field of burn care, qualitative evidence on how patients perceive and value online scar-related content remains limited. Therefore, the goal of this study was to investigate patients’ preferences regarding scar-related information, using MyScarSpecialst.com as a case example.

## 2. Methods

### 2.1. Study Design

This study used a qualitative descriptive research design which aims to describe experiences from the patients’ perspective and to gain a comprehensive understanding of their experiences [17]. Consequently, the analysis is more categorical than other methods [e.g., phenomenology and grounded theory] that involve high-level interpretation [17]. Focus groups were used to explore patient preferences regarding online information about scars and analyzed using thematic analysis.

### 2.2. Study Participants and Setting

Study participants were recruited from Oscare, an outpatient aftercare facility for persons with burns or scars of different origins (such as post-operative scars, elective surgery). Participants having scars were eligible to participate in the focus groups, provided they were 18 years or older and were proficient in Dutch. Participants with scars from self-harm, stretch marks, dermatological scars, unable to provide informed consent, and cognitive or intellectual impairment that affected oral communication, were excluded. The participants (see Table 1) were recruited orally by healthcare providers working at Oscare or students involved in this study. A convenience sampling strategy was used in this study (related to proximity, availability, and willingness to participate).

In preparation of the focus group, participants were instructed to visit the website ‘MyScarSpecialist’ and fill out a questionnaire regarding accessibility, comprehensibility, usability, design and completeness of the health information provided. Using MyScarSpecialist as a case example helped in collecting data about people’s needs and preferences related to online scar information.

### 2.3. Data Collection

Face-to-face, semi-structured focus groups were conducted after participants were instructed to visit MyScarSpecialist. The interview guide was not modified across the three focus groups. The wording of the questions was open, such as ‘what was your first impression when you visited the website’ and ‘what kind of information did you find interesting’. The three focus groups were moderated by three male healthcare professionals affiliated to Oscare with a background in physical therapy, nursing, or psychology. The focus groups were audio-recorded, transcribed verbatim, and lasted on average 130 min [range: 109–151 min].

### 2.4. Data Analysis

The manuscripts were imported into MAXQDA 24 software and inductively analyzed using thematic analysis, aligning with Braun and Clarke’s [18] approach. Two researchers (NVL and JM) independently analyzed the first two focus groups. The third focus group was analyzed by one researcher (NVL). Meaningful text fragments were coded, and codes were grouped into higher categories. Throughout the analytic process of defining themes, all authors engaged in three meetings (two after the first study phase and one after the third focus group; the latter also included students). The final themes were determined with the full research team during the last meeting.

### 2.5. Rigor

Several strategies were used to enhance trustworthiness. Data triangulation was pursued through the involvement of researchers with diverse disciplinary backgrounds and research or clinical experience. Two researchers had an academic background in physiotherapy (JM) or nursing and psychology (NVL). Both are experienced researchers, one of whom also has experience in qualitative research, and both had no treatment relationship with any of the participants. One researcher is an experienced clinician in physiotherapy (PM) and one researcher had a background in psychology (KM). The variation in perspectives and backgrounds enhances the credibility of the results. Reflexivity was pursued through self-reflection and discussions of differing perspectives during the meetings in a safe environment. Representative quotes were used to support the assessment of credibility and transferability. A third focus group was conducted to assess data saturation. No new themes emerged from this focus group, indicating data saturation. Finally, prolonged engagement in the burn care field and familiarity with the patient population facilitate trust-building with participants and support a deeper understanding of the study context. We believe this enhances the richness of the data.

### 2.6. Ethical Aspects

This study was approved by the ethical committee of ‘Ziekenhuis Netwerk Antwerp (ZNA)’ (now ‘Ziekenhuis aan de Stroom’ ZAS/‘Hospital by the River’) with EC number 5811. Informed consent was obtained from every participant for agreement to participate in this study.

## 3. Results

### 3.1. Participants Characteristics

A total of three focus groups were conducted, comprising 19 participants. Table 1 presents the participants’ characteristics. The participants had scars resulting from burns, trauma, or surgery, and all had a Caucasian skin type. Two focus groups were held in November and December 2023 at Oscare and the third one was held in December 2024. Participants participating in the first two focus groups had burn scars or scars caused by another trauma and one person participated as a non-patient participant. Three healthcare providers (a psychologist, physical therapist, and nurse) also attended one of the focus groups but their comments were beyond the scope of this paper and not included in the analysis. A subgroup (N = 3) of the participants had a prior treatment relationship with one of the moderators. The rationale to conduct a third focus group was to assess data saturation. Seven persons with scars of different origins participated in this focus group, predominantly post-operative scars. A researcher and three students attended the focus group as observers. There was no treatment relationship with any of the participants.

### 3.2. Themes

**Figure 1:**
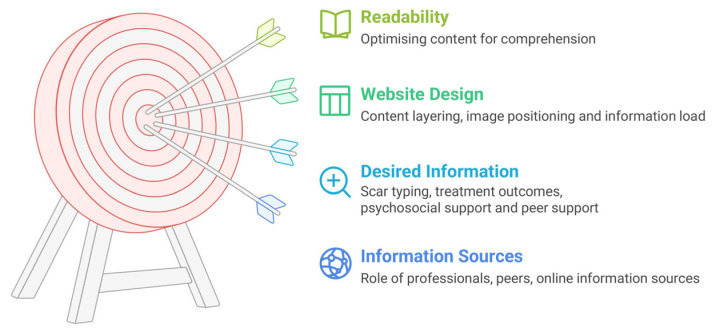
Focus group themes and subthemes.

The three focus groups yielded four themes (Figure 1): (1) Information Sources: The Role of Professionals, Peers, and Digital Media in information sharing; (2) Desired information: From scar typing to treatment outcomes to psychosocial impact; (3) Website design: Audience preferences on content layering, information load, and image positioning; (4) Readability: Optimizing content for comprehension. 

#### 3.2.1. Information Sources: The Role of Professionals, Peers, and Digital Media in Information Sharing

Participants generally prefer information from healthcare professionals because they find it efficient, trustworthy, and personalized. They can inquire and obtain responses that serve their best interests. Some participants mentioned that they when they had received sufficient information, they were less likely to seek additional information online. However, other participants also mentioned that healthcare providers are not constantly available and have limited time available, which may be a reason to look for other sources of information, provided that this information is reliable and originates from a validated source. It was also mentioned that a combination of professional information and an online search was likely.

“*For me, it was mostly the doctors, nurses, psychologists, and physios who gave me a lot of info. What was nice about it was that whenever you had a question about what was possible, you’d get an answer right away. That felt really efficient to me.*”

(focus group 1)

“*I’d probably ask the surgeon or someone like that: what do you think is best for a scar like this, what kind of treatment should I try? And then I’d also just look it up online.*”

(focus group 3)

Another important source of information that was highlighted was information from peers, although it may not be the most important source, according to a participant. Communication with peers occurred through social media and in-person interactions, providing useful tips.

“*You can really see that here at Oscare [aftercare centre], people got a lot out of talking to others who went through the same thing.*”

(focus group 1)

“*I did get a lot out of that [peer support], but to say it was the most important thing for me… not really.*”

(focus group 1)

Although there was generally a preference for information from healthcare professionals, several participants often sought information from online sources. Many participants noted that finding the desired information was challenging because it often lacked specificity, felt overwhelming, was not customized to their circumstances, or instilled fear of the worst outcomes that showed to be unrealistic with the passage of time.

“*When you look online, you get tons of info about burns in general, […] and that means you often end up reading all these worst-case scenarios […], so you don’t really get a realistic picture of what things will look like years later. Back then, I thought my scars would be way worse than they actually are now, eight years on.*”

(focus group 3)

“*Because online, there’s just so much info that it kind of overwhelms you. I don’t think it’s easy to search for something specific there.*”

(focus group 1)

Reliable and customized online sources of information were therefore found to be highly needed. Regarding children and adolescents, the participants recommended creating reliable information in various forms and for other platforms such as TikTok and Instagram.

#### 3.2.2. Desired Information: From Scar Typing to Treatment Outcomes to Psychosocial Impact

Participants were asked what information they needed regarding scars. On the website, scars were categorized into seven distinct types (in an earlier version for the participants at the first two focus groups): hypertrophic scars, burn scars, surgical scars, atrophic scars, vertical keloids, horizontal keloids, and small keloids. However, this classification was not always easily understood by all users. Consolidating the categories into four broader types in an adapted version of the website, i.e., burn-related, surgical, trauma-related, and dermatological scars, was found a more accessible and manageable framework. Concerning treatment options, several participants expressed that they were particularly interested in the outcomes of (laser) treatments, the level of pain it would cause, and the number of sessions required. Participants generally agreed that the underlying mechanisms of treatments were of lesser importance.

“*What I wanted to know as a patient about the laser treatment was that it would improve my skin’s elasticity and also help with the pigment and color. I wasn’t too concerned about the technical details of how it worked. What I really wanted to know in advance was how painful it would be.*”

(focus group 2)

Many participants were also interested in receiving more treatment advice. One participant mentioned that particularly about donor sites, there is limited information available. Information about creams and moisturizers, when and where to apply them, and whether they are freely available without a prescription was generally found important. Moreover, instructions about how to treat scars, illustrated with videos, were mentioned as useful.

“*How do I take care of my skin after a skin graft? […] I couldn’t find any information about the area where the skin was taken from.*”

(focus group 2)

“*People often think, ‘I’d like to try that cream too,’ so it’s helpful to know whether it’s available over-the-counter or only with a prescription.*”

(focus group 2)

Also, information about the costs, reimbursement, and practical information, such as where to find a specialist care provider, would be appreciated.

Several participants also advocated to provide information about the psychological impact of scars. Depending on the etiology of the scars [e.g., participants with burns and cancer were more likely to receive psychological care], it was mentioned they were inadequately prepared to engage with scars in the outside world.

“*The first time you go outside, you feel like everyone’s staring at you, like they’ve noticed the big cut on your neck—which, of course, isn’t really true, looking back.*”

(focus group 3)

Several participants valued peer support and mentioned that information regarding peer support was deemed a significant enhancement within the context of psychological support, and it was found essential that the website includes details on how to contact and interact with peers.

“*Even during the years of my rehab, I noticed that peer support is really important for a lot of patients. It’s important for me too, so maybe it’s fair to make a point of mentioning that on the website.*”

(focus group 3)

#### 3.2.3. Website Design: Audience Preferences on Content Layering, Information Load, and Image Positioning

Generally, the homepage should be attractive, inviting, and convey a positive message, and the purpose of the website should be immediately clear. Participants indicated that photos of scars do not need to appear immediately on the homepage.

“*When I visit the site […], I don’t want to be confronted with the scar right away. I’d prefer to see a more positive message, something like, ‘It’s going to be okay.’*”

(focus group 3)

To facilitate understandability and attractiveness of the website, participants had diverse ideas and stated that a mixture of photos, animations, drawings, and videos could be helpful. Several participants found that photos of scars were found to be very illustrative, although they may be impressive. Participants agreed that regarding the visibility of photo series featuring scars, it was appreciated that they are not readily available and that it is optional to view additional photos. Drawings were proposed when some softening is needed. Videos were considered helpful in demonstrating care procedures as individuals have a clearer understanding of what to expect. Graphic animations were found to be particularly useful for children. Figures and graphs were found to be less appealing.

“*The photos aren’t shocking, but they do make an impression. I don’t deal with a lot of scars myself, but they still hit me.*”

(focus group 1)

“*Or maybe have something that says if you want to see more photos, just click somewhere or something?*”

(focus group 2)

“*I marked photos because they help explain things, and I picked drawings because I think they soften things up a bit.*”

(focus group 2)

“*It’s good to know what to expect beforehand, and videos help with that way more than just explanations. Then I know what I’m in for. […] It’s also more lively and clear.*”

(focus group 1)

It was also mentioned that all types of scars should be presented, including normal scars, instead of only concentrating on hypertrophic and keloid scars.

“*I actually miss seeing a ‘normal’ scar. These are all pathological scars. Maybe some people with a ‘normal’ scar will look and wonder, does that mean my scar doesn’t need treatment?*”

(focus group 2)

The navigation options and icons that assist in the navigation process should be clear, including the meaning of the icons representing scar characteristics, which affects the sufficiency of the searches.

“*Those little icons showing scar features, I don’t think many people really connect them to scars. Maybe it’d be better to make a collage of photos showing different features? I think that would give a clearer idea of what’s meant.*”

(focus group 2)

“*Sometimes they use icons, sometimes photos. I’d rather see them together.*”

(focus group 2)

#### 3.2.4. Readability: Optimizing Content for Comprehension

Among the most important aspects of a website for patients is the understandability. Many participants stated that everyday language should be used, the information should not contain medical terminology or should be explained when unavoidable [using pop-ups], and the starting point should be that patients have no prior knowledge of scars. Participants explicitly stated that ‘scars’ is a subject that is little known to the public. Therefore, it is important to provide basic knowledge in simple language. Since the type of scarring is directing the search on the website in order to find the appropriate treatments, a participant suggested using simpler language to differentiate between the scars.

“*I think from your expert point of view, you sometimes assume everyone has a basic understanding, but that’s not always the case. […] I think you really need to start super simple and easy.*”

(focus group 1)

“*Scars, I don’t think a lot of people really know much about them.*”

(focus group 3)

“*Maybe you could add a step before that with easier-to-understand categories. Like, I have a burn, or I have a surgical scar.*”

(focus group 1)

It was also stipulated that the information on the website needs to be up-to-date, realistic, and reliable, and the text on the screen should be easy to read, which requires a sufficiently large font size.

## 4. Discussion

This study explored how people with scars perceived and preferred the presentation of online scar-related information, using MyScarSpecialist as a case example. Four overarching themes emerged: information sources, desired information (treatment options outcomes, and psychological impact), website design, and readability.

Participants used multiple information sources, including healthcare professionals, peers, and online information sources. For most, healthcare professionals were the main information providers. Peer interactions also provided valuable insights. However, an increasing number of patients sought additional information online. Earlier studies found that those with older injuries and parents of pediatric patients were more likely to use online resources [19]. This trend may reflect a growing need for additional sources during longer-term recovery. More recently, it was reported that burn survivors seek online information in all stages of recovery [20]. Notably, our study found it difficult to find personalized information in terms of treatment options and scar outcomes, supporting other study findings [20,21]. They were confronted with severe outcomes, without knowing how those outcomes related to their own scar. These findings highlight the demand for reliable and individualized online information. As Jones et al. [21] noted, healthcare professionals can play a valuable role in helping and guiding patients towards relevant and credible online resources.

Patients emphasized the need for clear explanations of scar types, treatment options, and expected outcomes. Understanding scar types was considered essential, as this formed the basis for other information on MyScarSpecialist. Presenting four scar types was found to be manageable when supported by clear text and visual illustrations. Preferences regarding laser treatment information and expected outcomes focused on visible improvements in skin characteristics and practical details, such as the treatment duration and session numbers. Adverse effects, such as pain, were also considered important informational elements. These findings are in line with Duchin et al. [22], who found that burn survivors desired far more detailed information and guidance on pain management and recovery, including sleep, pain medication, alternative therapies, plans for tapering analgesics and addiction risks. Incorporating this type of information into online resources could substantially enhance the quality of existing content and address key information gaps as reported by Manasyan et al. [12].

Participants additionally indicated a need for more information regarding the potential psychological impact of living with scars and peer support. Recent studies indicate that there are many barriers for seeking psychological support [23]. Although awareness of psychological care has grown, many barriers to seeking support remain [24]. Consequently, online information may play an important role in facilitating access to psychological support. The beneficial role of peer support has been consistently demonstrated among patients with burns [20,25], yet remains underused, particularly in groups of patients with smaller burns and a lower education level [26]. Participants and prior studies pointed to the need for more internet-based access points that connect individuals to appropriate peer networks. Online information may help patients discover, connect, and engage with peers.

The website’s ’attractiveness’, including visual appeal and conveying a positive message, emerged as one of the most critical elements. Participants suggested using a mix of photographs, illustrations, animations, or videos, depending on the context. This perspective aligns with user-centered design principles, which emphasize involving patients in design to improve acceptance and effectiveness of eHealth tools [21]. The use of photo galleries depicting scars was ambiguous. On the one hand, participants unanimously agreed that such images were helpful and necessary. For example, a series of photos depicting various types of scars was received positively. On the other hand, they emphasized that these should not be displayed on the homepage, but rather made visible on specific webpages. Although photographs may not be frequently used in online patient information—for example, only one of nine websites on low-back pain used photographs [27]—the use of photo galleries is more common in plastic surgery resources [28]. Especially concerning scar-related online information, the use of photographs seems quite relevant, but their use should be carefully considered. In certain circumstances, drawings or animations may be preferable. Applying co-design with end-users, iterative usability testing, and accessibility standards is likely to refine MyScarSpecialist.com and increase its value as an educational tool for patients with scars.

Readability was a crucial yet challenging aspect to attain. This study found that language, particularly in sections describing scar types and medical terminology, were far too advanced. Simplifying wording in these areas was seen essential for ensuring accessibility and comprehensibility. Similar concerns have been reported in multiple healthcare areas indicating, where online patient education often exceeds the recommended six-grade reading level [29,30]. For patients with lower health literacy, this raises the risk of misunderstanding and poorer health outcomes, according to Berkman et al. [31]. Daraz et al. [32] stated that poor readability can lead to misinformation and may have a harmful effect on health. Online scar-related information has also been shown to have low readability scores [12]. Tools such as the PEMAT instrument and artificial intelligence language models can help improve readability [33,34].

For example, a recent study demonstrated that rewriting online burn education texts using an AI language model substantially improved the targeted reading level (from just 4% to 18% after revision) [35]. Ensuring that MyScarSpecialist’s content adheres to health literacy best practices, including testing reading grade level and user feedback to simplify confusing sections, will be important going forward.

This study has some limitations that merit note. First, in two focus groups, a co-moderator had previously treated some participants, which might have influenced them to provide socially desirable responses. However, given the small number of such cases and the openness observed during the different focus group discussions, this risk is considered limited. Second, as focus groups are preferably homogeneous [36], the participation of healthcare providers in two focus groups might have affected group dynamics. Third, all researchers were affiliated with the organization that developed the website, which may have introduced bias. Nonetheless, the study was primarily conducted with a genuine interest to improve the website and align it more closely with patients’ needs, rather than seeking confirmation of existing content. On this basis, we consider it unlikely that the credibility of the findings was compromised. One of the strengths of this study is the inclusion of participants with a variety of scar types, enhancing the generalizability and practical applicability of the results. Using MyScarSpecialist as a case example allowed participants to interact with real content and provide nuanced, experience-based insights. This study also positions patients as co-developers, rather than passive end-users. This aligns with the Person-Based Approach [iterative qualitative insight to shape content, tone and features] [37] and with the ISO 9241-210 [38] human-centered design standard [International Organization for Standardization. ISO 9241-210:2019 Ergonomics of human-system interaction—Part 210: Human-centered design for interactive systems; ISO: Geneva, 2019], which are both linked to higher acceptability and engagement of eHealth tools. Comparable projects often rely on surveys or expert inspection alone, which miss lived-experience nuances.

The findings of this study underscore the critical role healthcare professionals play in guiding patients through the complex landscape of scar-related information. In both acute and aftercare settings, clinicians should proactively provide clear, tailored education about scar types, treatment options, expected outcomes, and potential psychological impacts. This includes not only verbal communication but also written and digital resources that match the patient’s literacy level and emotional readiness. Given the challenges patients face in finding personalized and trustworthy online information, healthcare workers should consider integrating structured educational moments throughout care pathways. Patients’ needs are not static, could change over time, and can be influenced by various factors. Providing information in the hospital during outpatient follow-ups or rehabilitation sessions, referring patients to reliable educational websites, and helping them interpret online content can reduce misinformation and anxiety.

Moreover, clinicians should actively promote peer support networks, both local and national, as they offer valuable psychosocial support and lived-experience insights. Embedding these networks into discharge planning or rehabilitation programs may enhance coping, reduce isolation, and improve long-term outcomes. Ultimately, a more structured, empathetic, and patient-centered approach to education and information delivery is essential to meet the evolving needs of burn survivors.

Finally, including patients as active partners in website design and in designing healthcare in general, as pointed out by Mesko et al. [13], reflects a broader shift toward patient-centered scar care [15]. Patient and population involvement in scar care is on the rise. Co-designed strategies, supported by principles of participatory and human-centered design, can strengthen trust, engagement, and educational impact across diverse care settings.

## 5. Conclusions

In conclusion, this study provides comprehensive insights into the perceptions of patients regarding our scar website. Identified themes and captured perspectives serve as a roadmap for optimizing scar websites and we hope it inspires all stakeholders of the scar community to go for co-creation with patients as partners in the development of (information on) scar care.

### Key Messages

1. Patients rely on a combination of healthcare professionals, peers, and digital media for scar-related information, yet report substantial difficulty finding trustworthy, personalized, and comprehensible online content.
2. Clear explanations of scar types, realistic treatment outcomes, practical care instructions, and information on peer and psychological support represent major unmet informational needs among individuals with scars.
3. Users prefer online scar information that is positively framed, visually balanced, and thoughtfully layered—offering optional access to scar photographs, supplemented by drawings, animations, and videos to support understanding.
4. Readability remains a critical barrier: patients strongly advocate for plain language, minimal jargon, and content designed for individuals with no prior knowledge of scars, underscoring the need for health-literacy-aligned materials.
5. Co-creation with patients meaningfully enhances the relevance, accessibility, and acceptability of digital scar information tools; integrating patient perspectives should be considered essential in developing future educational resources.

## Figures and Tables

**Table 1:** Demographics participants focus groups MyScarSpecialist.

Number	Participant Role	Age	Gender	Skin Type	Scar Cause	Location	Time Since Injury *
1	Patient	45	F	Caucasian	Acid	Face, Trunk, Arms	5
2	Patient	36	M	Caucasian	Burn	Face, Trunk, Hand	6
3	Patient	51	F	Caucasian	Trauma	Legs, Foot	2
4	Patient	33	M	Caucasian	Trauma	Leg, Foot	3
5	Non-patient participant	69	F	Caucasian	N/A	N/A	N/A
6	Patient	32	F	Caucasian	Burn	Face, Trunk, Arms, Hands, Legs	16
7	Patient	46	F	Caucasian	Burn	Face, Neck, Trunk	5
8	Patient	37	M	Caucasian	Burn	Face, Trunk, Hand	7
9	Patient	34	M	Caucasian	Trauma	Foot	4
10	Patient	78	F	Caucasian	Surgery	Trunk, Shoulder	50
11	Patient	54	F	Caucasian	Surgery	Neck	10
12	Patient	29	F	Caucasian	Burn	Legs	8
13	Patient	71	M	Caucasian	Surgery	Trunk	14
14	Patient	29	M	Caucasian	Burn	Face, Trunk, Arms, Hands, Legs	3
15	Patient	23	F	Caucasian	Surgery	Trunk	13
16	Patient	75	F	Caucasian	Surgery	Trunk	6

F: Female; M: Male; N/A: Not applicable; * Time since injury in years.

## Data Availability

The data contains potentially identifying and sensitive information, so due to privacy or ethical restrictions, they are not publicly available.
